# Visceral Adiposity Is Associated with Elevated Interleukin-1 Receptor Antagonist Levels and Anxiety Symptoms in Schizophrenia

**DOI:** 10.3390/ijms27146351

**Published:** 2026-07-17

**Authors:** Aleksandra Julia Oracz, Stefan Modzelewski, Mateusz Zwierz, Maria Suprunowicz, Joanna Matowicka-Karna, Napoleon Waszkiewicz

**Affiliations:** 1 Department of Psychiatry, Medical University of Bialystok, pl. Wołodyjowskiego 2, 15-272 Białystok, Poland; stefan.modzelewski@sd.umb.edu.pl (S.M.); zwierzmateusz@wp.pl (M.Z.);; 2 Clinical Laboratory Diagnostics Facility, Medical University of Białystok, pl. Waszyngtona 15A, 15-269 Białystok, Poland

**Keywords:** schizophrenia, visceral adiposity, cytokines, interleukin-1 receptor antagonist protein, anxiety symptoms, inflammation

## Abstract

Schizophrenia spectrum disorders are associated with visceral obesity and chronic low-grade inflammation. However, the role of inflammatory mediators in anxiety and depressive symptoms remains unclear. This study aimed to investigate associations between body fat distribution, circulating inflammatory mediators, and affective symptoms in schizophrenia. In this cross-sectional study, 67 patients with schizophrenia were assessed using the Positive and Negative Syndrome Scale (PANSS), with anxiety (G2) and depression (G6) items analyzed separately. Body composition, including visceral fat area (VFA), percent body fat (PBF), and skeletal muscle mass (SMM), was measured using bioelectrical impedance analysis. Serum concentrations of 38 immune mediators were determined using Multiplex technology. Multivariable regression analyses were performed to identify predictors of affective symptoms. Among all analyzed immune mediators, only interleukin-1 receptor antagonist (IL-1ra) was consistently associated with adiposity measures, showing the strongest correlation with VFA (rho = 0.53; *p* < 0.001). Higher VFA was independently associated with greater anxiety severity (β = 0.32; *p* = 0.017), whereas depressive symptom severity was associated with higher PBF (β = 0.32) and younger age (β = −0.25). Visceral adiposity was associated with anxiety severity in schizophrenia and may represent a potential marker warranting further investigation. The observed association between IL-1ra and adiposity supports a potential immunometabolic link between metabolic status and affective symptoms in this population.

## 1. Introduction

Schizophrenia spectrum disorders are among the most severe and burdensome psychiatric conditions. They pose a major clinical challenge not only because of their complex psychopathology but also due to the substantially increased risk of comorbid somatic diseases, particularly metabolic disorders. Patients with this diagnosis frequently exhibit higher rates of overweight, obesity, and metabolic syndrome (MetS), which may directly contribute to a reduction in life expectancy of 15–20 years compared with the general population [1,2]. Although this phenomenon is partly driven by lifestyle factors and the adverse effects of antipsychotic medications, a growing body of evidence suggests that the relationship between schizophrenia and metabolic dysregulation extends beyond purely iatrogenic effects [2].

In recent years, increasing attention has been directed toward the role of inflammatory processes in the pathophysiology of schizophrenia. A growing number of studies indicate that immune system dysregulation and chronic inflammation may play a significant role in the manifestation and progression of psychopathological symptoms [3]. Obesity, particularly the accumulation of visceral adipose tissue (currently recognized as a state of chronic low-grade inflammation), is no longer viewed merely as a passive energy storage compartment. Rather, adipose tissue functions as a highly active endocrine and immunological organ [4]. Excessive adipocyte hypertrophy leads to cellular hypoxia and extensive macrophage infiltration, accompanied by polarization toward the pro-inflammatory M1 phenotype [5], thereby inducing a systemic chronic low-grade inflammatory state [6]. This process is characterized by disrupted immune homeostasis, particularly a shift in T-cell responses toward the pro-inflammatory Th1 pathway at the expense of the anti-inflammatory Th2 response [7]. Notably, a similar imbalance and hyperactivation of the Th1 pathway have been observed during acute phases of schizophrenia [8], suggesting the existence of a common axis linking metabolic disturbances with central nervous system functioning.

Although neuroinflammation is commonly associated with the severity of both positive and negative symptoms of schizophrenia [9,10], particular attention has recently been paid to inflammatory mediators capable of crossing the blood–brain barrier and directly modulating neuronal circuits [11]. Clinically, these mechanisms may manifest as increased anxiety and depressive symptoms [12,13]. While classical pro-inflammatory markers such as interleukin-6 (IL-6) and tumor necrosis factor-alpha (TNF-α) are well-established components of this inflammatory cascade, one less frequently investigated mediator is the endogenous interleukin-1 receptor antagonist (IL-1ra). As a natural inhibitor of IL-1 signaling, elevated IL-1ra concentrations under conditions of heightened inflammation may reflect the activation of compensatory anti-inflammatory mechanisms. Previous studies have suggested that IL-1ra is associated with both obesity and metabolic disturbances; however, its role in schizophrenia and its relationship with symptom severity remain unclear [14,15].

Despite the growing evidence supporting the existence of an immunometabolic axis in schizophrenia, findings regarding its influence on depressive and anxiety symptoms remain inconsistent [16]. One reason for this inconsistency is that previous studies have generally examined these domains separately. Investigations focusing on immune profiling using Multiplex technology have typically limited metabolic assessment to the relatively nonspecific body mass index (BMI) [17], whereas studies employing precise measurements of adipose tissue distribution have usually been restricted to basic inflammatory markers such as C-reactive protein (CRP) [18]. The lack of integrated analyses combining objective measures of visceral adiposity with comprehensive cytokine profiling—and examining how the resulting inflammatory axis directly influences the expression of affective symptoms—represents one of the most significant gaps in current knowledge regarding the pathophysiology of schizophrenia.

To address this gap, the present study aimed to comprehensively evaluate the relationships between precise indicators of body fat accumulation, with particular emphasis on visceral fat area (VFA), a broad spectrum of inflammatory biomarkers, and the severity of anxiety and depressive symptoms in patients with schizophrenia. Unlike previous studies, the present project employed advanced multiparametric Multiplex technology, enabling the simultaneous mapping of a wide network of immune mediators. The primary hypothesis was that greater visceral obesity would be positively associated with markers of chronic inflammation and that this inflammatory state would act as an independent factor modulating and exacerbating coexisting mood disturbances and anxiety symptoms in the studied population.

## 2. Results

### 2.1. Baseline Characteristics and Metabolic Profile of the Study Cohort

A total of 67 patients with a diagnosis of schizophrenia were enrolled in the study. The mean age of the participants was 41.75 years (range: 18–69 years), and 64% of the cohort were male. Analysis of anthropometric parameters revealed a mean body mass index (BMI) of 27.82 ± 6.20 kg/m^2^. Notably, 36% of participants met the criteria for obesity (BMI ≥ 30 kg/m^2^), with a maximum recorded BMI of 48.20 kg/m^2^.

The mean percentage body fat (PBF) was 27.23 ± 12.12%, with a maximum value of 55.70%, while the mean visceral fat area (VFA) was 112.39 ± 61.50 cm^2^. Mean skeletal muscle mass (SMM) was 33.53 ± 7.29 kg.

Regarding psychopathological status, the mean severity scores for anxiety (PANSS item G2) and depression (PANSS item G6) were 2.24 ± 1.19 and 1.89 ± 1.18, respectively.

The Shapiro–Wilk test was statistically significant for the majority of analyzed variables, indicating deviations from normality. Furthermore, several variables demonstrated substantial distributional asymmetry (skewness > |1|). These findings justified the use of non-parametric statistical methods in subsequent correlation analyses.

Detailed descriptive statistics for all analyzed variables are presented in Table 1.

### 2.2. Associations Between Metabolic Parameters and Immunological Profile

**Figure 1:**
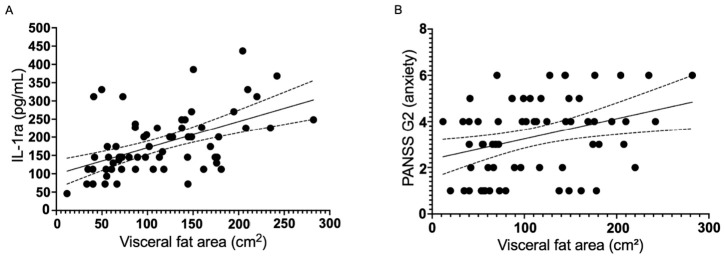
Associations of visceral fat area (VFA) with immunometabolic and clinical measures in patients with schizophrenia. (**A**) Positive correlation between VFA and serum Interleukin-1 receptor antagonist (IL-1ra) levels. (**B**) Positive association between VFA and anxiety severity, assessed by the PANSS G2 item. Solid lines represent the linear regression fit, while dashed lines indicate the 95% confidence intervals.

The correlation analysis revealed that, among all evaluated biomarkers, IL-1ra exhibited the strongest associations with body composition parameters. Statistically significant positive correlations were observed between IL-1ra levels and BMI (ρ = 0.52, *p* < 0.001), BFM (ρ = 0.52, *p* < 0.001), and VFA (ρ = 0.53, *p* < 0.001) (Figure 1A). The association with PBF was also positive and statistically significant, although somewhat weaker in magnitude (ρ = 0.48, *p* < 0.01).

In contrast to IL-1ra, the remaining biomarkers demonstrated only isolated correlations of relatively low magnitude. SDF-1α showed positive correlations with PBF (ρ = 0.27, *p* < 0.05) and VFA (ρ = 0.25, *p* < 0.05), and a negative correlation with SMM (ρ = −0.26, *p* < 0.05). Significant negative correlations with SMM were also observed for IL-17 (ρ = −0.25, *p* < 0.05) and β-NGF (ρ = −0.29, *p* < 0.05), whereas TRAIL was positively correlated with SMM (ρ = 0.26, *p* < 0.05). MCP-1 exhibited a positive correlation exclusively with BMI (ρ = 0.28, *p* < 0.05). However, following adjustment for multiple comparisons using the Benjamini–Hochberg False Discovery Rate (FDR) procedure, only the associations involving IL-1ra remained statistically significant.

Due to technical and laboratory-related issues, IL-1ra concentration data were unavailable for one participant; therefore, analyses involving this biomarker were performed on a sample of 66 participants (n = 66). A complete overview of the immunometabolic correlations is presented in Table 2. Importantly, after controlling for multiple comparisons using the Benjamini–Hochberg False Discovery Rate procedure, only associations involving IL-1ra remained statistically significant, underscoring the robustness of the IL-1ra–adiposity axis observed in the present study.

### 2.3. Correlations Between Adiposity Indices and Psychiatric Symptom Severity

Analysis of the relationships between somatic and psychiatric parameters revealed that the largest number of statistically significant associations involved anxiety severity, as measured by the G2 item of the PANSS. Positive correlations of moderate magnitude were observed between G2 scores and BMI (r = 0.30, *p* < 0.05), BFM (r = 0.35, *p* < 0.01), VFA (r = 0.32, *p* < 0.01), and PBF (r = 0.32, *p* < 0.01) (Figure 1B).

In contrast, depressive symptom severity (G6) demonstrated only a weak positive correlation with PBF (r = 0.26, *p* < 0.05). No statistically significant associations were identified between the remaining psychological variables and the analyzed metabolic parameters. Likewise, SMM was not significantly correlated with any of the psychological measures. A complete summary of these findings is presented in Table 3.

### 2.4. Linear Regression Models Examining Factors Associated with Anxiety and Depression Severity

A series of three linear regression analyses was conducted to determine whether BMI, PBF, and VFA were associated with anxiety severity (PANSS G2) after controlling for age and sex. All tested models reached statistical significance. The model including BMI explained 8.8% of the variance in anxiety scores (F(3, 62) = 3.10, *p* = 0.033, adjusted R^2^ = 0.088), with BMI emerging as the only variable significantly associated with anxiety severity (β = 0.29, t = 2.32, *p* = 0.024) (Table 4). Similarly, the model incorporating PBF accounted for 9.1% of the variance in anxiety (F(3, 62) = 3.17, *p* = 0.030, adjusted R^2^ = 0.091), and PBF was the sole significant predictor of G2 scores (β = 0.37, t = 2.36, *p* = 0.021) (Table 5). The highest proportion of explained variance (9.7%) was observed in the model including VFA (F(3, 62) = 3.32, *p* = 0.025, adjusted R^2^ = 0.097). In this model, VFA was the only variable significantly associated with anxiety severity (β = 0.32, t = 2.45, *p* = 0.017) (Table 6).

In a fourth regression analysis, the effects of sex, age, and PBF on depressive symptom severity (PANSS G6) were examined. The model was statistically significant and explained 8.7% of the variance in depression scores (F(3, 62) = 3.06, *p* = 0.034, adjusted R^2^ = 0.087). Within the model, both age and PBF were significantly associated with depressive symptom severity. However, the standardized regression coefficient was larger for PBF (β = 0.32, t = 2.05, *p* = 0.045) than for age (β = −0.25, t = −2.09, *p* = 0.041). Sex was not significantly associated with G6 scores (Table 7).

### 2.5. Exploratory Mediation Analysis

Exploratory causal mediation analyses were subsequently conducted with IL-1ra specified as the mediator. Across all four evaluated models, the natural indirect effect (NIE) did not reach statistical significance (all *p* > 0.20). Significant natural direct effects were observed for depressive symptoms in the PBF and VFA models and for anxiety symptoms in the VFA model. Detailed mediation results are presented in Supplementary Table S1.

## 3. Discussion

In contrast to some previous reports suggesting broad associations between obesity and inflammatory mediators in schizophrenia [19], the findings of the present study indicate a relatively selective pattern of correlations between adiposity-related parameters and the assessed immunological mediators within the investigated cohort. Despite the analysis of a comprehensive panel comprising 38 cytokines, chemokines, and growth factors, including IL-1α, IL-1β, IL-6, TNF-α, and IFN-γ, no statistically significant correlations were observed between the majority of these inflammatory mediators and the evaluated metabolic parameters or indices of adipose tissue distribution.

The principal finding of the present study was the identification of significant positive correlations between IL-1ra concentrations and all analyzed adiposity indices, with the strongest association observed for VFA. Importantly, after adjustment for multiple comparisons using the Benjamini–Hochberg False Discovery Rate procedure, only associations involving IL-1ra remained statistically significant, highlighting the robustness of the observed IL-1ra–adiposity relationship.

This observation is consistent with our initial hypothesis, formulated on the basis of previous evidence suggesting that pathological accumulation of visceral adipose tissue generates a pro-inflammatory milieu accompanied by a compensatory anti-inflammatory response [20,21]. Accordingly, the positive relationship between IL-1ra concentrations and VFA observed in our cohort may reflect the activation of such compensatory mechanisms in response to obesity-related inflammation.

Similar findings linking IL-1ra concentrations with adipose tissue accumulation and metabolic disturbances have been reported in studies involving patients with schizophrenia [22,23] and depression [24]. Lin et al. demonstrated that baseline IL-1ra levels in antipsychotic-naïve patients with schizophrenia were a strong predictor of subsequent fat accumulation during olanzapine treatment, as reflected by significant increases in leptin concentrations and the development of dyslipidemia [22]. This finding is particularly noteworthy given the well-established propensity of olanzapine to induce weight gain, especially central obesity [25]. Likewise, in an observational study conducted by Archer et al., obesity, assessed using BMI, emerged as the strongest factor associated with circulating IL-1ra concentrations among patients with depression. In their regression model, BMI accounted for 18.5% of the explained variance in IL-1ra levels [24].

Before adjustment for multiple comparisons, several weak associations involving SDF-1α, MCP-1, IL-17, β-NGF and TRAIL were observed. However, these correlations did not remain statistically significant after FDR correction and should therefore be interpreted cautiously and considered exploratory. Notably, these associations were primarily related to unfavorable alterations in body composition. In the analyzed cohort, higher SDF-1α concentrations were associated with both greater adiposity and lower skeletal muscle mass (SMM). This finding may suggest that the inflammatory processes identified in the present study are accompanied by adverse body composition remodeling toward sarcopenic obesity, characterized by simultaneous fat accumulation and muscle loss. However, this observation requires confirmation in prospective studies.

Given the scarcity of research examining the relationship between chemokines and muscle mass in psychiatric populations, these findings may represent an important avenue for future investigation. Although this phenomenon has not previously been explored in schizophrenia, our observations are supported by findings reported by Chen et al. in a geriatric population [26]. The authors demonstrated a significant inverse association between SDF-1α (CXCL12) concentrations and skeletal muscle mass and identified elevated SDF-1α levels as an independent risk factor for sarcopenia [26]. Furthermore, they showed that chronically increased concentrations of this chemokine may inhibit the myogenic differentiation of muscle stem cells [26,27]. Importantly, although adiposity-related indices were included in their analyses, no direct correlation between SDF-1α concentrations and body fat content was observed [26]. Although biologically plausible, the observed associations involving SDF-1α should be interpreted as hypothesis-generating, as they did not remain significant after adjustment for multiple comparisons.

It should also be emphasized that the increased susceptibility of patients with schizophrenia to early muscle loss and the development of sarcopenia [28], as well as sarcopenic obesity [29,30], have already been well documented. Depending on the population studied, sarcopenia may affect more than one-third of individuals with schizophrenia [28], whereas sarcopenic obesity has been estimated to occur in nearly 20% of patients [29,30]. In contrast to the apparent involvement of SDF-1α in muscle–fat compartment remodeling, the association observed for MCP-1 was limited exclusively to BMI and did not extend to specific adipose tissue depots. This finding is consistent with previous reports positioning MCP-1 primarily as a marker of generalized weight burden rather than a biomarker reflecting specific metabolic or lipid abnormalities [31]. Additional support for this interpretation comes from the study by Beumer et al., who demonstrated that although circulating MCP-1 (CCL2) levels increased in patients with schizophrenia and metabolic syndrome, BMI itself was not an independent determinant of MCP-1 concentrations in multivariable analyses [32]. Taken together, these findings may indicate that MCP-1 reflects broader aspects of adiposity, whereas IL-1ra appears to be more closely associated with visceral fat accumulation in the studied cohort.

While leading immunometabolic hypotheses in psychiatry link peripheral inflammation primarily to excess visceral adipose tissue [33]—a concept partially supported by the observed association between VFA and IL-1ra in the present study—our findings also point toward potential relationships involving skeletal muscle mass. Nominally significant correlations with SMM were observed for IL-17, β-NGF and TRAIL; however, these associations did not survive correction for multiple testing and should therefore be considered exploratory.

One possible explanation for these findings may lie in the distinct immunometabolic functions of skeletal muscle and adipose tissue that have been described previously in the literature. According to current mechanistic models, skeletal muscle exerts anti-inflammatory effects through the secretion of myokines during contraction, thereby counteracting the pro-inflammatory activity of adipose tissue [34,35,36]. This concept is supported by population-based studies conducted by Ying et al., in which progressive muscle loss and the development of sarcopenia were accompanied by significantly elevated circulating IL-17A concentrations [37]. These findings are consistent with the inverse association between IL-17 and SMM observed in the present study. It should be noted, however, that Sobiś et al. reported positive correlations between IL-17A concentrations and both BMI and body fat percentage (BF%) in patients with schizophrenia [38], findings that appear to contradict our results. This discrepancy may be largely attributable to important methodological limitations of the cited study. Specifically, BF% was not measured directly but estimated using mathematical formulas derived from the relatively nonspecific BMI, and the reported correlation was observed in an extremely small subgroup of female participants (n = 7) [38]. These limitations should be carefully considered when interpreting those findings. In contrast, body composition parameters in the present study were assessed directly using bioelectrical impedance analysis (BIA), allowing for a more detailed characterization of the study population.

In contrast to the potentially catabolic role of IL-17, the findings of the present study also highlight the involvement of two potentially protective mediators, β-NGF and TRAIL. The observed inverse correlation between β-NGF and skeletal muscle mass suggests a possible relationship between this neurotrophin and body composition parameters. However, the direction and biological significance of this association remain unclear and require further investigation. Experimental animal studies conducted by Jun et al. indicate that NGF may exert protective and regenerative effects on skeletal muscle under conditions of obesity. Specifically, the authors demonstrated that during obesity-associated skeletal muscle atrophy, NGF promotes cellular regeneration and suppresses pathways involved in muscle fiber degradation, including the myostatin signaling pathway [39]. Similarly, the complete absence of associations between TRAIL and adiposity-related indices, combined with its positive correlation with SMM, may suggest that TRAIL is linked to greater muscle mass in the studied cohort. This interpretation is supported by previous studies demonstrating positive associations between circulating TRAIL concentrations and preserved skeletal muscle mass [40]. Furthermore, TRAIL signaling has been shown to actively promote myogenesis while suppressing the expression of genes involved in obesity-related muscle atrophy [41]. Taken together, these observations suggest that β-NGF and TRAIL may be associated with skeletal muscle-related parameters in patients with schizophrenia. However, given the cross-sectional nature of the present study, neither the directionality nor the underlying mechanisms of these relationships can be determined. Nevertheless, the findings underscore the need for further research investigating the role of these mediators in body composition disturbances within this clinical population.

In addition to the biochemical analyses, the present study examined the direct relationship between body composition parameters and the mental health status of patients with schizophrenia, with particular emphasis on anxiety (G2) and depression (G6) severity. Our findings demonstrated that adiposity-related indices (BMI, PBF and especially VFA) were independently associated with affective symptom severity. Specifically, VFA emerged as the primary statistically significant predictor of anxiety symptoms, whereas depressive symptom severity was independently associated with higher PBF and younger age.

Importantly, despite exhibiting a distinct immunological profile, SMM was not significantly associated with any psychological variables. This finding may suggest that adiposity-related parameters are more closely linked to affective symptom severity than skeletal muscle mass. Interestingly, Pu et al. reported that among patients with stable schizophrenia, impaired muscle-related parameters, assessed using handgrip strength as a key clinical marker of sarcopenia, were strongly associated with substantially higher total PANSS scores and significant cognitive impairment [42]. In contrast to the findings of Pu et al., SMM was not associated with affective symptom severity in the present study. This discrepancy may be attributable to differences in the parameters assessed, as the cited study focused on muscle function measured by handgrip strength, whereas our investigation evaluated only skeletal muscle mass using BIA. Collectively, these findings suggest that structural and functional aspects of the muscular system may exhibit distinct relationships with the clinical presentation of schizophrenia, warranting further investigation.

It is noteworthy that the direct correlation analyses revealed body fat mass (BFM), expressed as the absolute amount of stored adipose tissue in kilograms, to be the parameter most strongly associated with primary anxiety (G2) among all body composition measures examined. However, when age and sex were simultaneously controlled for in regression analyses, VFA remained a significant independent predictor of anxiety severity and demonstrated the most robust association with anxiety symptoms among the analyzed adiposity indices.

An important contrast to our findings is provided by Sahin et al., who reported no significant relationship between visceral fat indices and anxiety severity in patients with primary anxiety disorders [43]. This discrepancy may indicate that the relationship between visceral adiposity and anxiety symptoms is disease-specific and may play a different role in schizophrenia than in populations with primary anxiety disorders.

The importance of VFA becomes even more apparent when comparing the regression models directly. A progressive increase in adjusted R^2^ values was observed from the model incorporating BMI to the model including VFA. Specifically, as the precision of adiposity assessment increased—from the general BMI measure (8.8% explained variance), through PBF (9.1%), to VFA (9.7%)—the predictive capacity of the models for anxiety severity improved accordingly. This observation is consistent with the correlation analyses, in which the strongest associations with IL-1ra were likewise observed for VFA. These findings highlight the well-recognized limitations of BMI [44,45,46] and suggest that a more detailed assessment of adipose tissue distribution may provide additional insights into the relationships among body composition, immunological markers, and affective symptoms in schizophrenia. Nevertheless, further studies are required to establish the clinical significance of these observations.

Importantly, neither metabolic parameters nor adiposity indices were significantly associated with the level of negative affect in the present cohort. This finding may suggest that the links between body fat accumulation and psychopathology are more pronounced for anxiety and depressive symptoms than for negative symptoms. One possible explanation is that negative symptoms of schizophrenia may be driven by distinct neurobiological mechanisms, potentially involving adipokines such as leptin, which are secreted in excess by adipose tissue in individuals with increased body weight [47]. Another noteworthy aspect of the present findings is the highly specific nature of the observed affective associations. Anxiety was analyzed using two complementary approaches: as the isolated PANSS G2 item (representing a pure anxiety dimension) and as a composite variable termed “anxiety level,” defined as the sum of PANSS items G1 + G2 + G3 + G4 + G6. The correlation analyses revealed a striking contrast between these approaches. While the isolated anxiety dimension (G2) demonstrated strong and statistically significant associations with adiposity-related parameters, including VFA, BMI, PBF, and BFM, the broader “anxiety level” variable failed to reach statistical significance.

This observation suggests that different operationalizations of affective symptoms may yield substantially different results. More specifically, aggregating heterogeneous symptoms into broad composite indices may obscure clinically meaningful and biologically specific relationships. Similar conclusions can be drawn from the study by Wang et al., in which affective status was assessed using a broad depressive factor calculated as the sum of PANSS items G2 + G3 + G6. Using this generalized approach, the authors found no significant psychopathological differences between obese and non-obese patients with schizophrenia [48]. Taken together, the comparison of these studies suggests that the analysis of more homogeneous psychopathological domains may facilitate the detection of associations that remain undetected when broader summary measures are employed.

The regression model constructed for depressive symptoms demonstrated that higher depression severity was predicted not only by increased body fat content (PBF) but also by younger age. The observation that anxiety was most strongly associated with VFA, whereas depression showed a closer relationship with PBF, suggests an interesting phenomenon. While anxiety symptoms appear to be more directly linked to the pro-inflammatory activity of visceral adipose tissue, depression in this patient population may involve an additional and substantial psychosocial component [49]. The strong association between PBF and depressive comorbidity in schizophrenia is supported by findings from other independent studies [49,50]. Avan et al. reported that the presence of metabolic disturbances and obesity increased the risk of developing a full depressive episode by more than threefold among patients with schizophrenia. However, isolated abdominal obesity, representing the clinical equivalent of elevated VFA, did not significantly distinguish patients with depression from those without depressive symptoms [50]. Furthermore, the role of adipose tissue itself in the development of depression has been supported by Mendelian randomization studies, which demonstrated that absolute fat mass constitutes a direct causal risk factor for depression, whereas centrally distributed fat deposits do not appear to exert a stronger effect in this regard [49]. One possible explanation for this relationship involves psychosocial factors previously described in the literature, including stigma and reduced satisfaction with physical appearance. Taken together, previous evidence and the present findings suggest that younger patients with greater adiposity may represent a subgroup characterized by a higher burden of depressive symptoms. Existing literature indicates that weight gain and unfavorable alterations in body composition may contribute to poorer psychological well-being, increased social withdrawal, and greater severity of depressive symptoms [49,51]. It is possible that the observed association between younger age and higher depression severity reflects a complex interaction among metabolic, psychosocial, and neurobiological mechanisms involved in mood regulation, including serotonergic neurotransmission pathways [52]. However, the underlying mechanisms cannot be determined based on the cross-sectional data presented in this study. If replicated in larger cohorts, these findings may have implications for strategies aimed at reducing both metabolic and affective complications in patients with schizophrenia.

Importantly, although sex was included as a covariate in all regression models, it did not emerge as a significant predictor of either anxiety or depression severity. These findings suggest that the relationship between excessive visceral fat accumulation and affective symptom severity may occur independently of patient sex. This observation is particularly relevant in schizophrenia, a disorder characterized by well-established sex-related differences in symptom profiles and metabolic complications. Numerous studies have demonstrated that sex is often a major determinant of clinical vulnerability, a finding that remains evident in multivariable analyses [53,54]. Yan et al. demonstrated that women with schizophrenia exhibit not only a significantly higher prevalence of metabolic complications, including obesity, but also greater baseline severity of depressive symptoms as measured by the PANSS compared with men [53]. Similarly, studies employing detailed body composition assessment using BIA have shown that the tendency toward excessive fat accumulation accompanied by muscle loss differs substantially between men and women with schizophrenia [54]. In the present study, however, the association between visceral adiposity and affective symptom severity remained significant after adjustment for sex. This may suggest that immunometabolic mechanisms related to visceral obesity play an important role irrespective of the sex-related differences reported in the previous literature. Nevertheless, these findings should be interpreted with caution, as the lack of statistical significance for sex may partially reflect the limited sample size and the unequal distribution of male and female participants in the study cohort.

The discrepancies between our findings and previous reports may also be partly attributable to ethnic, environmental, and metabolic differences across populations. Notably, the studies in which sex strongly influenced metabolic and affective profiles were conducted in East Asian populations, specifically Japanese [54] and Chinese [53] cohorts. These populations are characterized by distinct genetically determined metabolic phenotypes, different patterns of adipose tissue distribution, and population-specific anthropometric definitions of obesity compared with the Caucasian population examined in the present study.

Collectively, these observations support the hypothesis that excessive secretory activity of visceral adipocytes represents a primary source of mediators capable of crossing the blood–brain barrier and subsequently modulating central nervous system (CNS) function, thereby contributing to the development of anxiety symptoms [55,56,57]. Within this framework, the IL-1ra pathway emerges as a particularly promising target for future mechanism-based therapeutic interventions and represents an important direction for subsequent prospective longitudinal research.

## 4. Materials and Methods

### 4.1. Study Design

This study was observational, cross-sectional, and pilot in nature. It was designed as an exploratory investigation aimed at in-depth phenotyping of the immunometabolic profile of patients diagnosed with schizophrenia. Given the primary objective of the study, namely the evaluation of immunometabolic relationships and the identification of factors associated with the severity of psychopathological symptoms, no healthy control group was included.

Patient recruitment was conducted at the Department of Psychiatry, Medical University of Białystok; the Dr. Stanisław Deresz Independent Public Psychiatric Health Care Center in Choroszcz; and the “Bram-Medica” Mental Health Outpatient Clinic in Białystok, Poland, between January and September 2025. All participants received antipsychotic treatment in accordance with routine clinical practice. Quantitative assessment of antipsychotic treatment burden was not included in the statistical analyses.

The study protocol underwent a detailed review and received formal approval from the Local Bioethics Committee of the Medical University of Białystok (approval no. APK.002.528.2024). Prior to enrollment, all participants provided voluntary written informed consent. The consent process explicitly covered all study procedures, including peripheral blood collection, non-invasive body composition assessment, and comprehensive clinical evaluation.

The inclusion criteria were: (1) age between 18 and 69 years and (2) a confirmed diagnosis of schizophrenia established according to the clinical criteria of the International Statistical Classification of Diseases and Related Health Problems, 10th Revision (ICD-10). A total of 67 adult patients were ultimately enrolled in the study, including 43 men and 24 women.

To minimize the influence of potential confounding factors on the assessed immunological biomarkers, rigorous exclusion criteria were applied. Participants were excluded if they presented with clinical or laboratory evidence of acute infection, were pregnant, were receiving corticosteroid therapy, immunosuppressive treatment, or immunomodulatory therapy, or had a diagnosed acute or chronic somatic disease with a documented inflammatory etiology, including autoimmune disorders.

**Figure 2:**
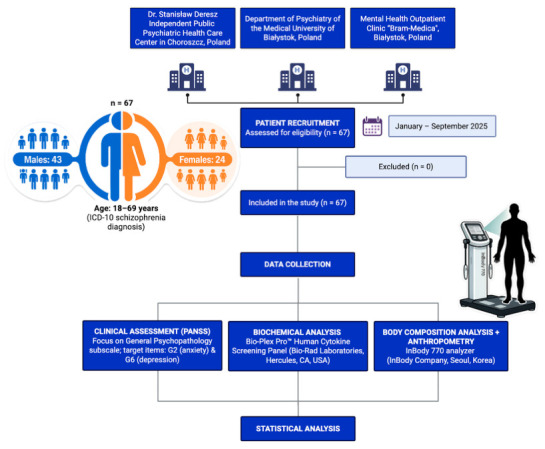
Flowchart of the study design detailing the multicenter recruitment of patients with schizophrenia and the sequential phases of clinical, biochemical, and body composition data acquisition. Abbreviations and symbols used in the figure: number of patients (n), Positive and Negative Syndrome Scale (PANSS), PANSS anxiety item (G2), PANSS depression item (G6).

The study’s design, participant enrollment, and data collection procedures are illustrated in a detailed flowchart in Figure 2.

### 4.2. Clinical Assessment

To objectively assess the current mental status and the severity of psychopathological symptoms, all participants underwent a comprehensive evaluation using the Positive and Negative Syndrome Scale (PANSS). All psychometric assessments were conducted in person by a single experienced psychiatrist. The use of a single-rater approach completely eliminated inter-rater variability and ensured optimal assessment consistency.

To maximize methodological rigor and maintain strict temporal consistency between biological markers and psychometric status, all study procedures were performed during a single visit under standardized clinical conditions. Clinical assessment in the form of a structured psychiatric interview was conducted on the same day as the morning body composition measurements and blood sample collection for immunological analyses.

Although the PANSS, the primary psychometric instrument used in this study, provides a comprehensive assessment of 30 symptoms, the present analysis focused primarily on two specific affective components within the General Psychopathology subscale: G2 (Anxiety) and G6 (Depression). The selection of these two items was intended to directly test the study hypothesis that chronic low-grade inflammation induced by visceral adiposity represents a key mechanism contributing to the modulation and exacerbation of coexisting mood and anxiety symptoms in patients with schizophrenia.

In addition, selected domain-based variables, calculated as the sum of scores assigned to predefined PANSS items, were included to provide a more comprehensive characterization of patients’ clinical status. Negative symptom severity was assessed using a variable termed “negative affect level,” calculated as the sum of PANSS Negative Subscale items N1–N7, encompassing blunted affect (N1), emotional withdrawal (N2), poor rapport (N3), passive/apathetic social withdrawal (N4), difficulty in abstract thinking (N5), lack of spontaneity and flow of conversation (N6), and stereotyped thinking (N7). Furthermore, an extended affective measure termed “anxiety level” was calculated as the sum of items G1 (Somatic Concern), G2 (Anxiety), G3 (Guilt Feelings), G4 (Tension), and G6 (Depression). These domain-based analyses were exploratory in nature and did not constitute primary study endpoints.

### 4.3. Body Composition Assessment

Body composition was assessed using a non-invasive bioelectrical impedance analysis (BIA) device, the InBody 770 analyzer (InBody Co., Seoul, Republic of Korea). The instrument provided measurements of body weight, percent body fat (PBF), body fat mass (BFM, kg), skeletal muscle mass (SMM, kg), and visceral fat area (VFA, cm^2^). In addition, standard anthropometric measurements, including height, waist circumference, and hip circumference, were obtained for each participant, and derived indices were subsequently calculated.

Body mass index (BMI) was calculated as body weight in kilograms divided by height in meters squared (kg/m^2^). For comparative analyses, participants were categorized according to BMI-based metabolic status. Consistent with established international guidelines, a BMI threshold of 25 kg/m^2^ was used. Participants with BMI < 25 kg/m^2^ were classified as having normal body weight (referred to as the normal-weight group), whereas those with BMI ≥ 25 kg/m^2^, encompassing both overweight and obesity, were classified as the overweight/obesity group.

All measurements and device calibration procedures were performed in strict accordance with the manufacturer’s recommendations. To ensure standardization, body composition analyses and anthropometric measurements were routinely conducted in the morning (between 7:00 and 9:00 a.m.) following overnight rest. Participants were examined in a fasting state and immediately after bladder emptying.

### 4.4. Biochemical and Immunological Measurements

Venous blood samples (5–15 mL) were collected from all participants during the morning hours, synchronized with the body composition assessment procedures. Blood was drawn using a closed vacuum aspiration system (S-Monovette^®^, Sarstedt, Nümbrecht, Germany) into serum-separation tubes containing a clot activator and into EDTA- or heparin-containing tubes for plasma collection. Blood collected into serum-separation tubes containing a clot activator was allowed to clot for approximately 30 min at room temperature prior to centrifugation, in accordance with routine laboratory procedures. EDTA- or heparin-containing tubes were processed shortly after collection. Following centrifugation, serum and plasma samples were aliquoted and stored at −80 °C until batch analysis.

Comprehensive quantitative profiling of inflammatory mediators and growth factors was performed using multiplex immunoassay technology. The commercially available Bio-Plex Pro™ Human Cytokine Screening Panel (Bio-Rad Laboratories, Hercules, CA, USA) was used, enabling the simultaneous quantification of multiple immune mediators within a single biological sample. The analyzed panel comprised 38 immunological mediators, including interleukins, chemokines, and growth factors. Among the assessed biomarkers were interleukin-1 receptor antagonist (IL-1ra), stromal cell-derived factor-1 alpha (SDF-1α), monocyte chemoattractant protein-1 (MCP-1), interleukin-17 (IL-17), beta-nerve growth factor (β-NGF), and tumor necrosis factor-related apoptosis-inducing ligand (TRAIL) (the complete list of analyzed mediators is presented in Table 1). All laboratory procedures were performed in strict accordance with the manufacturer’s protocol. Concentrations of all analyzed biomarkers and growth factors were expressed in picograms per milliliter (pg/mL).

### 4.5. Statistical Analysis

Statistical analyses were performed using IBM SPSS Statistics for Windows, Version 30 (IBM Corp., Armonk, NY, USA). Graphs and data visualizations were generated using GraphPad Prism for macOS, Version 10.5.0 (GraphPad Software, San Diego, CA, USA).

Initially, descriptive statistics were calculated, including the mean (M), median (Me), standard deviation (SD), skewness, kurtosis, and minimum and maximum values. The normality of quantitative variables was rigorously assessed using the Shapiro–Wilk test and skewness analysis. Because serum concentrations of inflammatory mediators and cytokines significantly deviated from a normal distribution and demonstrated substantial asymmetry (skewness > |1|), associations between these biomarkers and metabolic parameters were evaluated using Spearman’s rank correlation coefficient (ρ). In contrast, clinical variables derived from the PANSS and basic somatic parameters, whose distributions did not substantially violate the assumptions of parametric statistics, were analyzed using Pearson’s correlation coefficient (r).

One missing measurement of IL-1ra concentration was identified in the dataset due to technical laboratory issues encountered during Multiplex analysis. Consequently, this participant was excluded only from analyses directly involving IL-1ra, resulting in a sample size of n = 66 for analyses of this specific biomarker. All remaining clinical and biochemical data from this participant were retained in the other statistical analyses.

To identify variables independently associated with affective symptom severity (anxiety and depression), a series of linear regression analyses was conducted. BMI, PBF, and VFA were entered as independent variables, while age and sex were included as covariates. Prior to model interpretation, extensive diagnostic procedures were performed to verify the assumptions of linear regression. The absence of multicollinearity was confirmed using tolerance statistics and variance inflation factor (VIF) values, none of which exceeded 3.0. Furthermore, the assumptions of normally distributed residuals and homoscedasticity were verified. The potential influence of outlying observations on model stability and fit was assessed using Cook’s distance. Additionally, exploratory causal mediation analyses were performed with IL-1ra specified as the mediator. Detailed methodology is provided in the Supplementary Materials.

Statistical significance was set at *p* < 0.05 for all analyses. Results with *p*-values between 0.05 and 0.10 were interpreted as indicating a statistical trend. Because of the exploratory nature of the cytokine screening analyses, correction for multiple testing was performed using the Benjamini–Hochberg False Discovery Rate (FDR) procedure.

## 5. Conclusions

The present study sought to provide a comprehensive evaluation of immunometabolic relationships in patients with schizophrenia, with a particular focus on associations between body composition parameters, inflammatory mediators, and affective symptom dimensions. Following adjustment for multiple comparisons, IL-1ra emerged as the only inflammatory mediator consistently associated with adiposity-related parameters, particularly visceral fat area (VFA), suggesting that it may represent a marker of metabolic alterations accompanying visceral adiposity in schizophrenia rather than a direct indicator of clinical symptom severity.

Preliminary analyses indicated associations between adiposity-related measures and affective symptoms, particularly anxiety severity. However, these findings should be interpreted cautiously, as the observed relationships may be influenced by antipsychotic treatment, which was not controlled for in the present study.

Higher body fat percentage and younger age were associated with greater depressive symptom burden. At the same time, the results suggest the existence of a distinct biological pathway related to skeletal muscle mass (SMM), potentially involving β-NGF and TRAIL. Nevertheless, given that these associations did not remain significant after correction for multiple comparisons, they should be regarded as exploratory and hypothesis-generating observations.

Overall, the present findings suggest that IL-1ra may be more reflective of metabolic status than of clinical symptom severity itself. Although preliminary analyses indicated associations between adiposity-related measures and affective symptoms, these relationships may be influenced by antipsychotic treatment, which was not controlled for in the present study. Furthermore, exploratory mediation analyses did not support a pattern in which IL-1ra mediated the relationship between visceral adiposity and affective symptom severity. Therefore, these findings should be considered exploratory and hypothesis-generating. Future studies involving larger cohorts, detailed characterization of pharmacological treatment, and longitudinal designs are needed to better elucidate the complex relationships between adiposity, inflammation, and affective symptoms in schizophrenia.

### Study Limitations

Despite its innovative approach and the application of in-depth phenotyping, the findings of the present study should be interpreted in light of several important methodological limitations. First, the cross-sectional design precludes definitive conclusions regarding causal relationships between visceral fat accumulation, activation of inflammatory pathways, and the development of affective symptoms. Another limitation is the moderate sample size (n = 67) and the absence of a healthy control group. Although the sample was sufficiently large to construct linear regression models and identify the primary associations of interest, such as the IL-1ra–VFA axis, the lack of a reference group prevents assessment of the extent to which absolute biomarker concentrations differ from physiological norms, thereby limiting the broader generalizability of the findings. Furthermore, the moderate sample size resulted in several additional associations reaching only trend-level significance (0.05 < *p* < 0.10). This observation suggests that additional immunometabolic mechanisms may exist within the schizophrenia population but were not fully captured in the present study and might achieve statistical significance in larger cohorts.

With regard to measurement methodology, it should be noted that although bioelectrical impedance analysis (BIA) is a reliable and clinically useful tool, it represents an indirect approach compared with the current reference standards for visceral fat assessment, namely advanced imaging techniques such as magnetic resonance imaging (MRI) and dual-energy X-ray absorptiometry (DEXA). Similarly, although the PANSS remains the gold-standard instrument for psychopathological assessment in schizophrenia, the evaluation of anxiety and depression using single PANSS items (G2 and G6) does not fully capture the multidimensional nature of anxiety and depressive syndromes. Future studies would benefit from incorporating dedicated symptom-specific instruments, such as the Hamilton Depression Rating Scale (HAM-D) and the Hamilton Anxiety Rating Scale (HAM-A).

Another limitation that should be considered in the context of metabolic disturbances is the absence of classical laboratory measures, including lipid profile parameters, fasting glucose concentrations, and C-reactive protein (CRP) levels. Because the present study was designed to focus primarily on detailed immunological phenotyping using Multiplex technology and direct assessment of body composition, these conventional biochemical markers were not included in the study protocol. Future investigations should incorporate such measures to allow a more comprehensive characterization of the IL-1ra-related immunometabolic axis identified in this study within the framework of traditional metabolic syndrome (MetS) criteria.

Furthermore, anxiety and depressive symptoms were assessed using individual PANSS items (G2 and G6), which do not capture the full spectrum of affective symptomatology and should therefore be interpreted as dimensional indicators rather than comprehensive psychometric measures.

The most important limitation of the present study is the lack of a quantitative assessment of the impact of antipsychotic pharmacotherapy on the observed findings. Since antipsychotic medications, including olanzapine, clozapine, and quetiapine, may substantially influence metabolic parameters such as BMI and VFA, as well as inflammatory profiles [25], pharmacological treatment represents a significant potential confounding factor. In addition, the analyses did not include detailed clinical characteristics related to disease course, such as illness duration, age at onset, or number of psychiatric hospitalizations, all of which may influence immunometabolic relationships. Although the present cross-sectional study was designed to characterize the current clinical phenotype of patients under real-world conditions, future longitudinal investigations will be essential to disentangle the primary biological mechanisms of schizophrenia from the metabolic consequences of antipsychotic treatment.

## Figures and Tables

**Table 1:** Descriptive statistics of the analyzed variables and Shapiro–Wilk test results.

Variable	Mean	Median	SD	Skewness	Kurtosis	Min	Max	W	*p*
BMI	27.82	27.40	6.20	0.93	0.95	17.90	48.20	0.94	0.285
PBF	27.23	27.05	12.12	0.36	−0.56	6.00	55.70	0.94	0.277
BFM	23.71	22.00	13.47	0.98	1.20	4.40	70.50	0.93	0.203
VFA	112.39	100.45	61.50	0.58	−0.34	11.70	282.10	0.94	0.246
SMM	33.53	33.70	7.29	−0.16	−1.06	18.70	45.60	0.95	0.334
CTACK	519.17	514.36	187.00	0.56	0.20	202.88	1096.05	0.98	0.885
Eotaxin	134.38	115.30	77.55	1.25	1.31	33.61	387.38	0.83	**0.003**
Basic FGF	31.34	28.53	10.11	1.56	3.27	12.74	69.50	0.91	0.083
G-CSF	54.86	49.12	24.36	0.94	0.38	14.43	109.06	0.88	**0.017**
GRO-α	1676.62	1768.57	274.57	−1.65	2.74	702.65	2271.97	0.84	**0.004**
HGF	477.03	404.20	477.99	5.12	26.74	158.58	3084.16	0.45	**<0.001**
IFN-γ	46.86	42.97	16.02	0.73	0.26	18.73	86.70	0.88	**0.022**
IL-1α	20.48	17.89	7.93	0.95	1.81	4.59	45.38	0.93	0.198
IL-1β	3.54	3.43	0.99	1.00	1.77	1.71	7.12	0.95	0.393
IL-1ra	181.04	145.17	84.25	0.90	0.56	46.12	436.80	0.93	0.191
IL-2Rα	41.91	39.37	15.39	1.36	2.55	16.87	90.01	0.83	**0.003**
IL-3	0.67	0.46	0.47	1.31	1.55	0.17	2.14	0.88	**0.020**
IL-4	0.74	0.72	0.36	2.41	11.97	0.16	2.67	0.95	0.368
IL-6	2.98	2.34	2.26	2.27	7.94	0.37	13.86	0.95	0.396
IL-7	13.55	10.72	11.00	1.26	1.50	0.32	51.15	0.89	**0.033**
IL-8	7.59	6.36	4.85	2.72	8.02	3.12	28.64	0.73	**<0.001**
IL-9	607.95	601.51	127.30	1.29	4.01	321.44	1068.60	0.82	**0.001**
IL-12 (p40)	32.92	31.93	23.99	1.08	0.78	6.68	107.04	0.86	**0.011**
IL-16	24.48	22.36	11.80	1.81	4.12	8.94	68.83	0.81	**0.001**
IL-17	8.17	7.78	2.64	1.68	4.03	5.11	18.97	0.94	0.216
IL-18	44.11	42.67	13.80	0.05	−0.36	10.95	77.21	0.98	0.904
IP-10	324.16	270.79	234.74	2.26	5.98	62.44	1331.45	0.80	**0.001**
LIF	40.71	40.11	10.60	0.36	0.49	15.30	72.27	0.94	0.222
MCP-1	21.26	18.85	12.29	2.49	7.76	8.57	72.31	0.81	**0.001**
M-CSF	7.97	7.63	2.47	0.68	0.11	3.35	14.21	0.91	0.085
MIF	367.95	333.10	137.74	0.90	1.27	125.62	852.59	0.90	**0.041**
MIG	288.30	155.18	532.62	5.95	40.29	69.25	4065.35	0.42	**<0.001**
MIP-1α	1.43	1.28	0.61	0.96	0.49	0.40	3.04	0.93	0.209
MIP-1β	308.18	316.73	44.43	−1.11	3.22	152.67	433.19	0.93	0.180
β-NGF	2.33	2.20	1.31	3.26	15.84	0.19	9.38	0.58	**<0.001**
PDGF-BB	2035.94	2037.95	624.54	0.55	0.20	806.63	3879.04	0.96	0.485
RANTES	8990.41	9312.19	2831.32	−1.27	2.78	1128.19	14,789.50	0.84	**0.004**
SCF	43.96	40.87	14.88	2.14	7.63	25.86	115.99	0.95	0.465
SCGF-β	83,653.48	88,074.75	28,577.54	−0.50	−0.58	18,674.56	133,864.00	0.96	0.507
SDF-1α	1266.70	1201.29	310.55	1.00	2.49	531.26	2375.56	0.73	**<0.001**
TNF-α	135.01	132.87	31.42	0.48	2.08	49.76	245.19	0.92	0.121
TNF-β	498.20	508.07	78.73	−0.59	5.00	219.12	789.94	0.71	**<0.001**
TRAIL	39.81	40.14	8.45	0.42	0.90	20.82	66.59	0.94	0.269

Abbreviations and symbols: Standard Deviation (SD), Minimum value (Min), Maximum value (Max), Shapiro–Wilk test statistic (W), *p*-value for Shapiro–Wilk test (*p*), Body Mass Index (BMI), Percent Body Fat (PBF), Body Fat Mass (BFM), Visceral Fat Area (VFA), Skeletal Muscle Mass (SMM), Cutaneous T-cell-attracting chemokine (CTACK), Eotaxin/CCL11 (Eotaxin), Basic fibroblast growth factor (Basic FGF), Granulocyte colony-stimulating factor (G-CSF), Growth-regulated oncogene-alpha (GRO-α), Hepatocyte growth factor (HGF), Interferon-gamma (IFN-γ), Interleukin-1 alpha (IL-1α), Interleukin-1 beta (IL-1β), Interleukin-1 receptor antagonist (IL-1ra), Interleukin-2 receptor alpha (IL-2Rα), Interleukin-3 (IL-3), Interleukin-4 (IL-4), Interleukin-6 (IL-6), Interleukin-7 (IL-7), Interleukin-8 (IL-8), Interleukin-9 (IL-9), Interleukin-12 subunit p40 (IL-12 (p40)), Interleukin-16 (IL-16), Interleukin-17 (IL-17), Interleukin-18 (IL-18), Interferon-gamma-induced protein 10 (IP-10), Leukemia inhibitory factor (LIF), Monocyte chemoattractant protein-1 (MCP-1), Macrophage colony-stimulating factor (M-CSF), Macrophage migration inhibitory factor (MIF), Monokine induced by interferon-gamma (MIG), Macrophage inflammatory protein-1 alpha (MIP-1α), Macrophage inflammatory protein-1 beta (MIP-1β), Beta-nerve growth factor (β-NGF), Platelet-derived growth factor-BB (PDGF-BB), Regulated on Activation Normal T Cell Expressed and Secreted (RANTES), Stem cell factor (SCF), Stem cell growth factor-beta (SCGF-β), Stromal cell-derived factor-1 alpha (SDF-1α), Tumor necrosis factor-alpha (TNF-α), Tumor necrosis factor-beta (TNF-β), Tumor necrosis factor-related apoptosis-inducing ligand (TRAIL). All serum concentrations of immunological mediators and growth factors are expressed in picograms per milliliter (pg/mL). Units: BMI (kg/m^2^), PBF (%), BFM (kg), VFA (cm^2^), SMM (kg).

**Table 2:** Associations between metabolic parameters (BMI, VFA, BFM, PBF, SMM) and individual immunometabolic mediators in patients with schizophrenia. Due to multiple testing, a False Discovery Rate (FDR) correction according to the Benjamini–Hochberg procedure was applied.

Variable	BMI	PBF	BFM	VFA	SMM
CTACK	0.01	−0.10	−0.07	−0.07	0.00
Eotaxin	−0.09	−0.09	−0.10	−0.08	−0.06
Basic FGF	0.04	0.06	0.04	0.08	−0.04
G-CSF	−0.12	−0.05	−0.08	−0.02	−0.05
GRO-α	−0.07	−0.15	−0.12	−0.10	0.17
HGF	0.03	−0.05	0.00	0.03	0.10
IFN-γ	0.08	0.23	0.18	0.22	−0.19
IL-1α	0.03	0.12	0.06	0.10	−0.19
IL-1β	−0.10	−0.10	−0.12	−0.07	−0.02
IL-1ra	0.52 ***	0.48 **	0.52 ***	0.53 ***	−0.07
IL-2Rα	0.06	0.04	0.08	0.10	0.08
IL-3	0.05	0.18	0.12	0.16	−0.14
IL-4	0.04	0.10	0.06	0.07	−0.17
IL-6	0.10	0.19	0.14	0.15	−0.22
IL-7	0.06	0.16	0.12	0.14	−0.08
IL-8	0.09	0.02	0.04	0.06	0.00
IL-9	−0.12	−0.09	−0.12	−0.08	0.09
IL-12 (p40)	0.15	0.26	0.22	0.24	−0.26
IL-16	0.18	0.23	0.18	0.22	−0.14
IL-17	−0.02	0.14	0.06	0.10	−0.25
IL-18	0.07	0.13	0.14	0.17	−0.02
IP-10	0.16	0.17	0.19	0.23	−0.03
LIF	0.13	0.17	0.14	0.18	−0.01
MCP-1	0.28	0.14	0.22	0.23	0.09
M-CSF	0.11	0.20	0.17	0.20	−0.12
MIF	0.01	0.07	0.05	0.08	−0.10
MIG	0.09	0.09	0.10	0.14	0.00
MIP-1α	0.15	0.11	0.14	0.15	0.06
MIP-1β	−0.09	−0.01	−0.03	0.02	0.02
β-NGF	0.07	0.24	0.16	0.22	−0.29
PDGF-BB	0.05	0.15	0.12	0.17	−0.11
RANTES	0.01	0.20	0.12	0.18	−0.21
SCF	0.08	0.19	0.16	0.16	−0.10
SCGF-β	−0.07	−0.14	−0.10	−0.06	0.22
SDF-1α	0.14	0.27	0.21	0.25	−0.26
TNF-α	0.00	0.07	0.05	0.10	−0.03
TNF-β	−0.11	−0.04	−0.06	−0.03	0.06
TRAIL	−0.11	−0.23	−0.16	−0.14	0.26

Abbreviations and symbols: *** *p* < 0.001, ** *p* < 0.01, Body Mass Index (BMI), Percent Body Fat (PBF), Body Fat Mass (BFM), Visceral Fat Area (VFA), Skeletal Muscle Mass (SMM), Cutaneous T-cell-attracting chemokine (CTACK), Eotaxin/CCL11 (Eotaxin), Basic fibroblast growth factor (Basic FGF), Granulocyte colony-stimulating factor (G-CSF), Growth-regulated oncogene-alpha (GRO-α), Hepatocyte growth factor (HGF), Interferon-gamma (IFN-γ), Interleukin-1 alpha (IL-1α), Interleukin-1 beta (IL-1β), Interleukin-1 receptor antagonist (IL-1ra), Interleukin-2 receptor alpha (IL-2Rα), Interleukin-3 (IL-3), Interleukin-4 (IL-4), Interleukin-6 (IL-6), Interleukin-7 (IL-7), Interleukin-8 (IL-8), Interleukin-9 (IL-9), Interleukin-12 subunit p40 (IL-12 (p40)), Interleukin-16 (IL-16), Interleukin-17 (IL-17), Interleukin-18 (IL-18), Interferon-gamma-induced protein 10 (IP-10), Leukemia inhibitory factor (LIF), Monocyte chemoattractant protein-1 (MCP-1), Macrophage colony-stimulating factor (M-CSF), Macrophage migration inhibitory factor (MIF), Monokine induced by interferon-gamma (MIG), Macrophage inflammatory protein-1 alpha (MIP-1α), Macrophage inflammatory protein-1 beta (MIP-1β), Beta-nerve growth factor (β-NGF), Platelet-derived growth factor-BB (PDGF-BB), Regulated on Activation Normal T Cell Expressed and Secreted (RANTES), Stem cell factor (SCF), Stem cell growth factor-beta (SCGF-β), Stromal cell-derived factor-1 alpha (SDF-1α), Tumor necrosis factor-alpha (TNF-α), Tumor necrosis factor-beta (TNF-β), Tumor necrosis factor-related apoptosis-inducing ligand (TRAIL).

**Table 3:** Associations between metabolic parameters and the severity of depression, anxiety, and negative affect in patients with schizophrenia.

Variable	BMI	PBF	BFM	VFA	SMM
G6—Depression	0.15	0.26 *	0.21	0.21	−0.13
Negative affect level	−0.12	−0.05	−0.05	−0.03	0.00
G2—Anxiety	0.30 *	0.32 **	0.35 **	0.32 **	0.00
Anxiety level	0.20	0.24	0.24	0.23	−0.03

Abbreviations and symbols: ** *p* < 0.01, * *p* < 0.05, Body Mass Index (BMI), Percent Body Fat (PBF), Body Fat Mass (BFM), Visceral Fat Area (VFA), Skeletal Muscle Mass (SMM), Positive and Negative Syndrome Scale (PANSS).

**Table 4:** Linear regression model predicting anxiety severity based on sex, age, and BMI.

Variable	*B*	*SE*	*Beta*	*t*	*p*
(Constant)	2.51	1.23		2.04	**0.046**
Sex	−0.45	0.43	−0.13	−1.06	0.295
Age	−0.02	0.02	−0.16	−1.35	0.182
BMI	0.08	0.03	0.29	2.32	**0.024**

Model fit: F(3, 62) = 3.10, *p* = 0.033; R^2^adj = 0.088; N = 66. Abbreviations and symbols: *p*-value (*p*), unstandardized regression coefficient (B), standard error (SE), standardized regression coefficient (Beta), Student’s *t*-test statistic (t), adjusted R-squared (R^2^adj), Analysis of Variance F-statistic (F), Body Mass Index (BMI), Positive and Negative Syndrome Scale (PANSS).

**Table 5:** Linear regression model predicting anxiety severity based on sex, age, and PBF.

Variable	*B*	*SE*	*Beta*	*t*	*p*
(Constant)	3.02	1.07		2.83	**0.006**
Sex	0.12	0.54	0.04	0.23	0.822
Age	−0.03	0.02	−0.18	−1.52	0.135
PBF	0.05	0.02	0.37	2.36	**0.021**

Model fit: F(3, 62) = 3.17, *p* = 0.030; R^2^adj = 0.091; N = 66. Abbreviations and symbols: *p*-value (*p*), unstandardized regression coefficient (B), standard error (SE), standardized regression coefficient (Beta), Student’s *t*-test statistic (t), adjusted R-squared (R^2^adj), Analysis of Variance F-statistic (F), Percent Body Fat (PBF), Positive and Negative Syndrome Scale (PANSS).

**Table 6:** Linear regression model predicting anxiety severity based on sex, age, and VFA.

Variable	*B*	*SE*	*Beta*	*t*	*p*
(Constant)	3.60	0.92		3.91	**<0.001**
Sex	−0.23	0.45	−0.07	−0.51	0.615
Age	−0.03	0.02	−0.17	−1.47	0.148
VFA	0.01	0.00	0.32	2.45	**0.017**

Model fit: F(3, 62) = 3.32, *p* = 0.025; R^2^adj = 0.097; N = 66. Abbreviations and symbols: *p*-value (*p*), unstandardized regression coefficient (B), standard error (SE), standardized regression coefficient (Beta), Student’s *t*-test statistic (t), adjusted R-squared (R^2^adj), Analysis of Variance F-statistic (F), Visceral Fat Area (VFA), Positive and Negative Syndrome Scale (PANSS).

**Table 7:** Linear regression model predicting depression severity based on sex, age, and PBF.

Variable	*B*	*SE*	*Beta*	*t*	*p*
(Constant)	3.53	1.12		3.15	**0.002**
Sex	0.11	0.56	0.03	0.20	0.842
Age	−0.04	0.02	−0.25	−2.09	**0.041**
PBF	0.05	0.02	0.32	2.05	**0.045**

Model fit: F(3, 62) = 3.06, *p* = 0.034; R^2^adj = 0.087; N = 66. Abbreviations and symbols: *p*-value (*p*), unstandardized regression coefficient (B), standard error (SE), standardized regression coefficient (Beta), Student’s *t*-test statistic (t), adjusted R-squared (R^2^adj), Analysis of Variance F-statistic (F), Percent Body Fat (PBF), Positive and Negative Syndrome Scale (PANSS).

## Data Availability

The original contributions presented in this study are included in the article/Supplementary Material. Further inquiries can be directed to the corresponding author.

## Supplementary Materials

The following supporting information can be downloaded at: https://www.mdpi.com/article/10.3390/ijms27146351/s1.
