# Supplementary material for: Visceral Adiposity Is Associated with Elevated Interleukin-1 Receptor Antagonist Levels and Anxiety Symptoms in Schizophrenia
Source: Int J Mol Sci. 2026 Jul 17;27(14):6351. doi: 10.3390/ijms27146351 (PMC13410328; doi:10.3390/ijms27146351)
Supplement: Supplementary file 1 [file ijms-27-06351-s001.zip › ijms-4396214-supplementary.pdf]

Outcome	Exposure	NIE β (95% CI)	p	NDE β (95% CI)	p	TE β (95% CI)	p	Mediation
G2 Anxiety	PBF	0.040 (-0.672; 0.751)	0.913	1.514 (-0.129; 3.156)	0.071	1.553 (0.191; 2.915)	0.025	No
G6 Depression	PBF	-0.528 (-1.359; 0.302)	0.212	2.287 (0.781; 3.793)	0.003	1.758 (0.429; 3.087)	0.010	No
G2 Anxiety	VFA	-0.006 (-0.629; 0.617)	0.985	1.335 (0.043; 2.628)	0.043	1.329 (0.278; 2.381)	0.013	No
G6 Depression	VFA	-0.398 (-1.094; 0.298)	0.262	1.467 (0.313; 2.622)	0.013	1.069 (0.015; 2.123)	0.047	No

Supplementary Table S1. Exploratory causal mediation analysis evaluating IL-1ra as a potential mediator of the associations between adiposity measures (percent body fat [PBF] and visceral fat area [VFA]) and affective symptom severity after adjustment for age and sex.

Causal mediation analyses were performed in Stata 19 (StataCorp LLC, College Station, TX, USA) using the mediate command. IL-1ra was specified as the mediator, and all models were adjusted for age and sex. Because adiposity measures were analyzed as continuous exposures, causal effects were estimated by comparing the 10th and 90th percentiles of the exposure distribution (PBF: 13.0% vs. 46.6%; VFA: 40.2 vs. 204.4 cm^2). Natural indirect effects (NIE), natural direct effects (NDE), and total effects (TE) are presented as β coefficients with robust 95% confidence intervals.
